# Representations and coverage of non-English-speaking immigrants and multicultural issues in three major Australian health care publications

**DOI:** 10.1186/1743-8462-7-1

**Published:** 2010-01-03

**Authors:** Pamela W Garrett, Hugh G Dickson, Anna Klinken Whelan, Linda Whyte

**Affiliations:** 1 Simpson Centre for Health Services Research, University of New South Wales, 2-4 Speed St Liverpool, BC1871, Sydney, Australia; 2 Department of Ambulatory Medicine, Liverpool Health Service, Elizabeth Drive, Liverpool, BC 1871, Sydney Australia; 3 University of NSW, Faculty of Medicine, High St, Randwick, Sydney Australia; 4 School of Public Health and Community Medicine, University of NSW, High St Randwick, Sydney, Australia

## Abstract

**Background:**

No recent Australian studies or literature, provide evidence of the extent of coverage of multicultural health issues in Australian healthcare research. A series of systematic literature reviews in three major Australian healthcare journals were undertaken to discover the level, content, coverage and overall quality of research on multicultural health. Australian healthcare journals selected for the study were *The Medical Journal of Australia *(MJA), *The Australian Health Review *(AHR), and *The Australian and New Zealand Journal of Public Health *(ANZPH). Reviews were undertaken of the last twelve (12) years (1996-August 2008) of journal articles using six standard search terms: 'non-English-speaking', 'ethnic', 'migrant', 'immigrant', 'refugee' and 'multicultural'.

**Results:**

In total there were 4,146 articles published in these journals over the 12-year period. A total of 90 or 2.2% of the total articles were articles primarily based on multicultural issues. A further 62 articles contained a major or a moderate level of consideration of multicultural issues, and 107 had a minor mention.

**Conclusions:**

The quantum and range of multicultural health research and evidence required for equity in policy, services, interventions and implementation is limited and uneven. Most of the original multicultural health research articles focused on newly arrived refugees, asylum seekers, Vietnamese or South East Asian communities. While there is some seminal research in respect of these represented groups, there are other communities and health issues that are essentially invisible or unrepresented in research. The limited coverage and representation of multicultural populations in research studies has implications for evidence-based health and human services policy.

## Background

Mainstream healthcare research can be perceived as being neglectful of cross-cultural research. It is frequently seen as methodologically difficult to do with significant interpretative problems [1,2]. Resources may be inadequate for the translation of study instruments or the employment of bicultural researchers and interpreters. Concepts do not always have semantic or linguistic equivalence across languages or cultures [3,4]. Sampling methods, subject recruitment, achieving adequate sample sizes and representative samples may pose additional challenges [5,6]. However, it can equally be argued that to ignore populations with limited English proficiency may result in poor study validity and generalisabilty, could be considered discriminatory in culturally diverse social contexts, and, in a healthcare environment increasingly committed to evidence-based policy, may ultimately produce poor policy.

Representations of immigrants have shifted considerably in the period since the end of World War Two. Thirty years ago cross-cultural health researchers would have studied 'migrant patients', or, a little later, 'patients from non-English-speaking backgrounds'-at that time, a definable (constructed) field of study, which assumed that the similarities within these groups allowed them to be neatly categorised, labelled and understood as one entity. From the post-World War Two period to the 1960s, the presence of immigrants was basically ignored. They were expected to be invisible and assimilate into the dominant society as quickly and as fully as possible [7]. Research studies indicate that, in the 1960s, doctors and psychiatrists developed an interest in exotic migrant 'diseases' or pathologies and the 'culture-specific' health problems of migrants [8]. In the 1970s, the developing interest in social justice led to a desire to build social capital (including universal health insurance and migrant participation) to overcome the poverty and inequity experienced by, amongst others, migrant groups [9]. Lobby groups advocated for healthcare access and migrant rights in the 1980s [7,10]. Most recently, multicultural health services have operated as targeted strategies to address the specific needs of specific groups of people with limited English proficiency [11]. In other words, conceptions (and re-conceptions) of the field of immigrant health study are a product of history, are relational, and have associated socially constructed meanings.

Congruent with these shifts in constructions of immigrant health problems and issues has been a shift in the language for representing and describing immigrants. Immediately post-war, the official term for immigrants was 'new Australians', indicating a sense of differentness, of welcome, but also an expectation of assimilation. This changed to 'ethnic' or 'migrant' in the 1970s and to 'non-English speaking background' (NESB) in the 1980s and 1990s. These terms reflected the cultural pluralism that was current at the time, with a person's cultural and linguistic origins and migration status being viewed as constants, rather than evolving in dynamic interaction with the host country. In 1996, the federal government formally replace the term 'NESB'. *The House of Representatives Standing Committee on Community Affairs Inquiry into Migrant Access and Equity *[12] argued that being from a non-English-speaking background did not indicate disadvantage; an increasingly valid observation as the class and educational background of immigrants had shifted by that decade.

However, the agreed replacement term, 'culturally and linguistically diverse (CALD)' was taken up by federal authorities in Australia (but not unilaterally by the predominately Labor state government authorities). It asserted and highlighted the difference between immigrants and mainstream Australians, and emphasised culture, rather than language. The new term's emphasis on being different from the Australian cultural norm implied a marginalising attribute-a distinction from the majority, the Australian-born. This representational change was undertaken within a context where there was increasing national questioning about the ideology of multiculturalism, immigration levels, and contestation over what it meant to be Australian. The changing discourse on immigrants has frequently served to define non-immigrants as legitimate and immigrants as different, and therefore marginal, and potentially less legitimate: 'The Other' [13,14]. Interestingly, CALD has also come to convey a sense of being 'up-to-date' or current. The naming and re-naming of this population group by the state indicates continued attention to people who have migrated to Australia in the past several decades. The changing representations reflect the ongoing production and re-production of social understandings about immigrants.

No recent Australian studies or literature provide evidence of the extent of coverage of multicultural health issues in Australian healthcare research. A series of literature reviews in major healthcare journals were undertaken to discover the level, content, coverage and overall quality of research on multicultural health.

## Results

### Search Results: *Medical Journal of Australia*

In the MJA, over this 12-year period, there were 7,176 publications, of which 2,227 were articles. The Scopus search, using six search terms, yielded 80 articles, after exclusions, and the Medline search yielded 69 articles, after exclusions. Three hundred and fifty articles were accessed through the eMJA search. A further two articles accessed through the other two search strategies and not accessed by the eMJA search were added to those included in the study [15,16]. After exclusions (N = 205), 184 articles were studied in depth. Thirty-one of these were subsequently excluded as they contained no mention of multicultural health-related issues, leaving 153 MJA articles in the final analysis.

**Figure 1:**
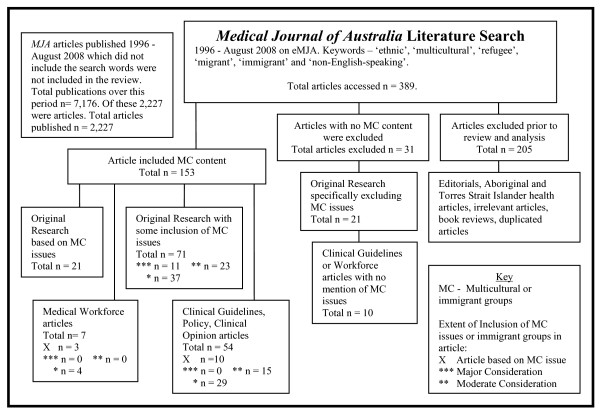
***Medical Journal of Australia *literature search method and results**.

Thirty-four original articles, or 1.5% of the articles published in the MJA over the 12 year period, directly related to multicultural health concerns. Twenty-one articles were original research articles, 10 more were policy issues, guidelines or case studies and three were related to medical workforce issues. Figure 1 outlines the strategy and results of the search.

The 31 articles which were excluded at this final stage are worthy of examination. There were 10 clinical guidelines or workforce articles that did not mention non-English-speaking populations or groups. A further 21 original research articles in the MJA explicitly excluded the participation of people with limited English. These articles, which knowingly excluded the participation of people with limited English proficiency, included articles concerning: women's understanding of their breast cancer diagnosis [17]; a prevalence study of domestic violence [18]; a random sample of young people to determine their ability to identify depression and psychosis [19]; a random sample testing the intentions of young people should they experience mental health problems [20]; a random sample of South Australian households to determine the extent of self-reported medication [21]; predictors of participation in screening programs or cancer tests [22,23]; pathways to care for cancer patients [24]; health status during treatments [25]; co-morbidity studies [26]; and, studies of post-operative or post-treatment complications [27,28]. Generally, these articles simply listed their exclusion in the methods section or in a section on the study's limitations. In explanation, one said that lack of funds for translators restricted the study to people who could speak English. Others noted that the questionnaire was in English only or that the study required consent to be signed in English. One study [29] did not provide the program of outpatient stabilisation of newly diagnosed Type 1 Diabetes patients to patients without English.

Of the 153 included articles, 21 were original research studies that specifically focused on immigrant groups. Five articles provided descriptive information on African or refugee medical and physical health derived from on-arrival screening or related services [30-34]; three discussed Vitamin D deficiency in veiled and dark-skinned women and their babies [35-37]; five were concerned with the mental health of refugees [15,38-41]; one with iron depletion in Arabic-speaking toddlers [42]; one examined the hospital utilisation of refugees based on their source country [43]; one examined the differential access rights of Temporary Permit Visa holders, as compared with refugees [44]; one studied the primary care utilisation patterns of 341 asylum seekers in Melbourne [45]; one studied the effectiveness of outpatient malarial treatment with African refugees [46]; one looked at the barriers to healthcare access for newly arrived African refugees [47]; one studied shared antenatal care by women of non-English speaking backgrounds [16]; and one studied the positive relationship between intravenous drug use and Human Immunodeficiency Virus in Indochinese communities [48].

A further 71 original MJA research articles took some account of patients with limited English, ethnic groups or multicultural health. These articles were further categorised as having a high (11 studies), moderate (23 studies) or minor (37 studies) treatment of multicultural health issues within the study.

The 11 original research articles categorised with a high level of consideration of multicultural health included, for example, a study showing that overseas-born men were more likely to commit domestic homicide and that NESB women in arranged marriages were particularly at risk [49]; a case study of an immigrant patient negatively affected by herbal medicine usage [50]; a study of the use of complementary medicines [51]; a study reporting mainstream community concerns about refugees bringing diseases to Australia [52]; a prevalence study of tuberculosis (TB) in Melbourne secondary students, which found that being born overseas was a predictor for contraction of TB [53]; an analysis of the differential death and disability risk factors in developed and developing countries [54]; a study of the efficacy of intramuscular cholecalciferol injection for Vitamin D deficiency [55]; and, a study finding non-English-speaking (NES) patients, in comparison with English-speaking patients, were more likely to consult a bilingual GP, attend a solo metropolitan practitioner and to consult a GP for respiratory, endocrine and digestive problems [56].

Examples of the 23 original research articles that were rated as having a moderate consideration of multicultural health included: a study of young women and risk taking, which found young women from non-English-speaking backgrounds (NESB) were more likely to be involved in car accidents [57]; a study of snorkelling deaths, which found 10 out of 27 were 'NESB tourists' [58]; a study of homicides during psychotic episodes, which found that 19% (an over-representation) were from a 'NES background' [59]; a study predicting deaths among young offenders, which found that having a drug-related offence was a major predictor and that the risk of drug-related offences was 13 times greater for 'Asian' young offenders and two times greater for those from other non-English-speaking backgrounds [60]; a study of cosmetic surgery and health status noting that NESB women were less likely to undergo cosmetic surgery [61]; a study of the spread of Hepatitis C, finding that Vietnamese migrants and prisoners were more likely to share needles [62]; a study of the help-seeking behaviour of men with erectile dysfunction finding that NESB men with this problem were less likely to seek help [63]; a study of gestational diabetes noting a general prevalence of 3.6% 'north east Asian' and 'south east Asian' women having prevalence rates of 13.7% and 12.5% respectively [64]; a study of obesity and overweight in an obstetric population noting increasingly high body mass index (BMI) associated with 'minority ethnic descent' [65]; a study of Type 2 Diabetes in youth finding 'ethnic' young people were over-represented [66]; and an epidemiological study and discussion of child health in Australia, which noted the particular vulnerability of asylum seekers and the problems of institutional racism [67].

Fifty-four MJA articles were clinical guidelines, policy issues, or case study articles rather than original research articles. Of these, 10 were categorised as articles concerned primarily with multicultural health-related issues, 15 as moderately inclusive and 29 as having a minor mention of multicultural health issues. Examples of articles concerned primarily concerned with multicultural health issues included: an article outlining the importance of cultural competence in dealing with adolescents [68] or in medical practice [69]; an argument for the introduction of RU-486 in Australia [70]; a discussion of the forced detention of non-compliant TB sufferers [71]; discussions about the usage of complementary and alternative medicines and the need to introduce its study into medical schools [72,73]; a case study of a neonate with high lead levels associated with the mother's ingestion of herbal remedies [74]; a discussion of the legal and ethical implications of medically enforced feeding of detained asylum seekers who are on hunger strike [75]; concerns about the limited rights to healthcare of asylum seekers [76,77]; and an article detailing the trauma experienced by children and their families held in detention [78].

Seven MJA articles related to medical workforce issues, mainly focused on the stresses associated with being a doctor [79-82]. Birrell et al. [83] discussed the lack of national standards and variable standards and knowledge of overseas trained doctors (OTDs); Arkles et al. [84] outlined similar issues associated with healthcare provided by OTDs in remote Aboriginal populations; McGrath [85] discussed the importance of integrating OTDs into the medical workforce. The latter three articles are considered to be based on multicultural health issues.

### Search Results: *Australian Health Review*

**Figure 2:**
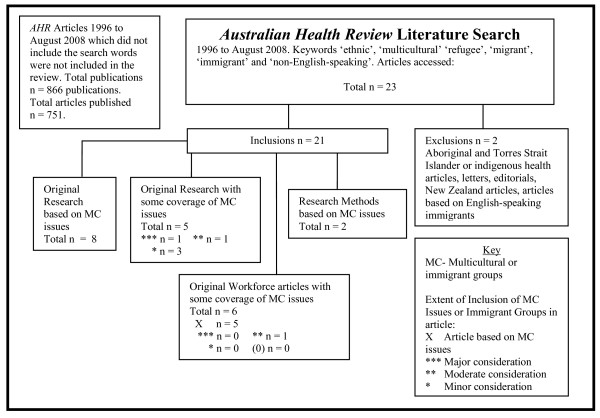
***Australian Health Review *literature search method and results**.

In the AHR over this 12-year period there were a total of 866 publications. Of these, 751 were articles. Eighteen articles relating to multicultural health issues were identified initially from the Medline and Scopus searches-two of these were excluded as they were New Zealand articles. A verifying manual search through the journal website yielded a further five articles [86-90]. Of these final 21 articles, eight were original research based on multicultural health, six were health workforce articles based on multicultural health issues and two were articles concerned with cross-cultural research methods. Fifteen original articles, or 1.9% of the articles published over the 12-year period, directly related to multicultural health concerns. One further workforce article had a moderate consideration of multicultural issues and three original research articles had a minor inclusion of multicultural health issues. Figure 2 outlines the AHR search strategy and the results of the search.

The AHR contained eight original research articles based on multicultural health issues. Tran et al. [91] profiled 829 clients of the Ethnic Obstetric Liaison Officer Service in the south west of Sydney and concluded that ethnic-specific service models are necessary for linguistic and cultural relevance; Heaney and Moreham [92] surveyed 109 hospital staff and found they reported under-usage of professional interpreters and inappropriate usage of family and friends as interpreters; Chan and Quine [93] conducted focus groups in Chinese to determine the health needs of this community; Han [94] explored the factors sustaining usage of herbal medicines by Koreans in Australia; Renzaho [89] negatively reviewed community service delivery to culturally and linguistically diverse populations and suggested a new model of needs-led 'cultural consultation' be implemented; Murray and Skull [88], discussed the barriers to care faced by refugees, including language, culture, legal, employment and policy barriers, and outlined available health and social resources and entitlements; Wen et al. [90] found culturally diverse populations were less likely to be visited by child health nurses or volunteers and more likely to find such visits 'uncomfortable'; and Strong et al. examined the health status of overseas born Australians and found lower reported mortality and hospital utilisation [95].

Six AHR articles related to healthcare workforce issues. Five of these workforce articles were primarily based on multicultural health-related issues. Tang et al. [96] studied the (negative) attitudes to nursing in secondary students from non-English-speaking background; Mathews et al. [97] differentiated between the role of bilingual staff and interpreters based on the views of a range of bilingual and interpreting staff; Johnson et al. [98] surveyed bilingual staff in an area health service and found a language 'mismatch' between bilingual staff and the local populations, and that bilingual staff mainly used their language in simple conversations; Mathews et al. [87] qualitatively evaluated the role of ethnic health staff in hospitals; and Bayram et al. [86] researched overseas trained doctors and found that, in comparison with locally trained doctors, they were younger, worked more sessions, were less experienced and saw a different range and mix of patients (newer patients, more disadvantaged and Indigenous patients). The AHR study, which considered multicultural health issues in a moderate way, looked at the changing workforce challenges and the changing profile of Australian medical students [99].

Two AHR articles related to multicultural health methods: one identified and analysed national health and welfare data collections and found that Australian Bureau of Statistics standards and classifications relating to ethnicity were quite widely used [100]; Whelan [101] outlined a process for the rapid appraisal of views of non-English-speaking clients.

Five articles were original research that considered multicultural health issues. One article [102], rated with a high level of inclusion of multicultural health issues, examined future directions for Victoria's maternity services and found 'immigrant' women were consistently less satisfied with their care. A moderate level of consideration of multicultural health issues was evident in an article that examined the use of respite services among carers of non-institutionalised people [103]. Three articles were original research articles that took multicultural health issues into account in a minor way [104-106].

### Search Results: *Australian and New Zealand Journal of Public Health*

There were 1,727 publications in the ANZJPH over this period. Of these, 1,168 were articles. The Scopus and Medline searches yielded 175 articles. After exclusions, a total of 110 articles were reviewed in full. Twenty-five reviewed articles contained no consideration of multicultural health issues, effectively leaving 85 articles-7.2% of the total articles published over the 12-year period.

There were 41 original research articles in the area of multicultural health in the ANZJPH over the 12 years. Of these, 16 (39%), were published in 2001 or more recently. A further 44 research articles contained some inclusion of multicultural health issues; four contained a major consideration, six a moderate mention and 34 articles contained a minor consideration of multicultural health issues.

**Figure 3:**
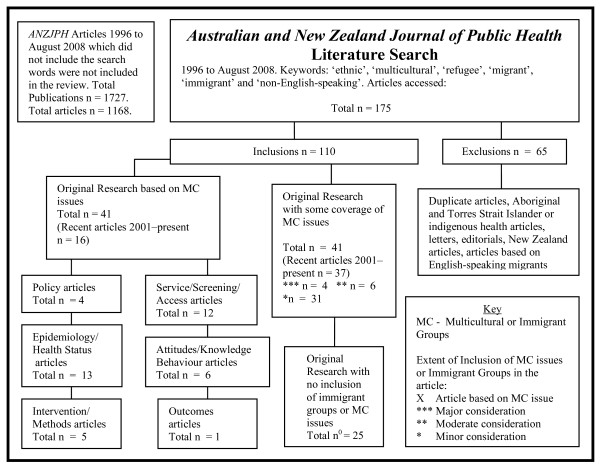
***Australian and New Zealand Journal of Public Health *literature search method and results**.

Of the original research articles, 13 original articles were concerned with epidemiology or health status issues. Pell et al. [107] studied demographic and work-related changes in 'Asian' female sex workers; Hellard et al. [108] found a high prevalence of blood borne viruses in 127 ethnic Vietnamese drug users; Steel et al. [109] found the psychiatric status of asylum seeking families accommodated in remote detention centres was very significantly compromised, and Mares and Jureidini [110] found very high levels of mood disturbance and post-traumatic stress symptoms in children and families accommodated in detention centres; Kingsford Smith and Szuster [111] measured dental health in refugees from the former Yugoslavia and Iraq and found it was considerably poorer than that of a matched group of social security recipients; Rissel et al. [112] surveyed 2,573 secondary students with large 'Arabic' and 'South East Asian contingents' and found delayed smoking uptake in these groups; Rissel et al. [113] found 49% of males and 29% of females in the Lebanese community smoked; Lin and Ward [114] studied the smoking-related habits and attitudes of 1,084 'ethnic Chinese'; Taylor et al. [115] found 'migrants' had lower rates of coronary heart disease probably related to selection, diet and lifestyle; Burton and Lancaster [116] profiled the Pacific Islanders' obstetric population and found three times the average rate of gestational diabetes, greater hypertension and higher perinatal mortality; Sullivan and Shepherd [117] studied the obstetric profile of Vietnamese-born women and found higher gestational diabetes, lower pre-eclampsia and lower birth weights; Rissel [118] developed an eight-item scale of acculturation for use in cross-cultural studies; Brown et al. [119] surveyed 198 Filipinas and found good self-perceived health status; Ireland and Giles [120] found an Italian-born group's blood pressure increased in comparison to a decline in the Australian-born group in a suburb in Melbourne.

Of the policy-related articles, two were concerned with the detention of asylum seekers [121,122], and another found poor implementation of ethnic health policy in community mental health centres [123].

Service- and screening-related original research findings included: lower reporting rates of pap test in women speaking a language other than English and varied uptake of pap test by region of birth [115]; low cervical screening rates for Vietnamese women was related to acculturation and years of residency in Australia [124]; translated personalised letters sent to Vietnamese women were ineffective in increasing cervical screening rates [119]; Hepatitis B status and vaccination coverage was studied in Vietnamese schoolchildren [125], in Vietnamese intravenous drug users [126], in infants from ethnic groups with high carrier prevalence [127], and in students at intensive English high schools [128]; Davis et al. [129] found NESB elderly aged care assessment clients were under-referred for assessments and were more likely to require nursing home placement; Dolman et al. [130] highlighted language barriers, cultural insensitivity and service knowledge as access issues for people with limited English; Davidson et al. [131] reviewed refugee access to dental services and found several barriers; Neale et al. [132] surveyed people recently arrived from the Horn of Africa and found 50% reported health access difficulties, mainly related to communication.

Two studies outlined major issues associated with achieving quality methods in cross-cultural research studies [5,6]; Mitchell et al. [133] found cervical screening rates increased with intensive ethnic media publicity; Page et al. [134] found no change in mammography screening attendance in Italian-born women as a result of ethnic media publicity; while Wong and Wang found that the participation rates in a Chinese survey were increased by using translated instruments [135].

Six studies discussed knowledge, attitudes and health-related behaviours. Telephone surveys of Vietnamese males' knowledge of sexually transmitted diseases and blood borne viruses found poor knowledge [136]; high levels of Hepatitis B and poor information about Hepatitis B was found in Lao and Cambodian groups [137]; Rice and Naksook [138] examined Thai women's perceptions about caesarean births; Cheek et al. [139] surveyed Vietnamese women's attitudes to cervical screening and found reasonably high participation rates and reliance on GPs and family for information; Plunkett and Quine [140] found carers with limited English were reluctant to institutionalise their elderly relatives in nursing homes; and Maneze et al. [141] described the pattern of kava use among 73 Tongan men in the Macarthur area of Sydney.

### Search Results: *Summary*

Table 1 summarises results of the analysis of these three journals. In total there were 4,146 articles published in these journals over the 12-year period. A total of 90, or 2.2% of the total articles, focused primarily on multicultural health issues. A further 62 articles had a major or moderate level of consideration of multicultural health issues and 107 had a minor mention. In total, 259, or 6.3% of all articles, included some mention of multicultural issues.

**Table 1:** Summary of Results of the Analysis of Three Journals

	Total number of articles in journal	Number of MC articles	MC articles as a % of total articles	Number of other articles with major or moderate consideration of MC issues	Number of other articles with minor consideration of MC issues
***MJA***	2,227	34	1.53	49	70
***AHR***	751	15	1.99	3	3
***ANZJPH***	1,168	41	3.51	10	34

**Total**	**4,146**	**90**	**2.17**	**62**	**107**

### Limitations

This study is limited by the accuracy of the search tools. The Medline and Scopus searches in each case yielded different articles. Despite attempts to overcome the shortfalls, relevant articles may not have been retrieved through this search strategy, just as some retrieved articles were clearly not relevant.

It is further acknowledged that a significant amount of Australian multicultural health research has focused on mental health. A literature review in the area of mental health has also demonstrated gaps in research [142]. A study such as this requires a level of judgment in respect of the level of consideration of multicultural issues included in a study. It is acknowledged that although the principal researcher checked the categorisation with another researcher, such judgments can always be debated.

## Discussion

Valuable insights can be gained from comparing the representations of immigrants in each of these three journals. Almost all the original MJA research articles focused on newly arrived refugees. Almost all focused on relatively exotic, if not ethnic-specific illnesses. Most (13 articles) related either to the mental or physical health of newly arrived refugees or asylum seekers, and five dealt with concerns about vitamin deficiencies in veiled or dark-skinned women and their babies, or in Arabic speaking toddlers. The MJA published seminal articles and opinion pieces in these areas particularly concerning the Australian policy and treatment of asylum seekers. Researchers often focus on the most recently arrived community as they are considered to be the most different from the mainstream [143]. Yet the representations remain very limited. Of the 2,227 articles published during the 12-year period, only 21 (0.9%) were original research and a total of 34 (1.5%) were concerned primarily with multicultural health issues. A further 71 MJA original research articles including some discussion of multicultural issues, with 11 articles discussing multicultural health issues as a major component of the article and 23 as a moderate component. In others (37 articles), the mention of diversity was incidental, or, what Minas et al. [142] described as 'tacked-on', or an after-thought to the main discussion. Of the 54 MJA articles categorised as policies, guidelines or case studies, 10 were primarily concerned with multicultural health issues.

There was a slightly larger number of original multicultural health articles (n = 41) featured in the ANZJPH compared with the MJA, representing 3.4% of the total articles published in the ANZJPH over the 12-year period. The majority of original ANZJPH articles were published prior to 2001, with only 16 original research articles published since 2001-possibly a reflection of a changing social climate. The range of health issues covered in the ANZJPH was somewhat limited, with seven concerned with blood borne viruses or sexually transmissible infections, five with cervical cancer and pap tests, three with obstetric profiles, three with smoking prevalence, two with dental health, one with coronary heart disease, one with blood pressure and one with the health of Asian sex workers. Six articles critically discussed ethical issues, health status or service implications of the policy of detaining asylum seekers and their children in remote detention centres.

The 15 original AHR research articles were diverse in subject matter. Five addressed workforce changes, recruitment issues, and bilingual staffing in hospitals. The other 10 articles addressed a range of issues related to epidemiology, access, service delivery, and cross-cultural research methodologies.

Apart from two AHR articles that assessed service access [124,131] it is interesting that few studies in these three journals have evaluated service quality or effectiveness. For example, surprisingly few studies have evaluated the impact, demand and cost effectiveness of healthcare interpreter services. Indeed, there is little Australian evidence regarding the most common methods of facilitating communication in healthcare with people who have limited English. Access studies have focused on community services and aged care services.

Wide-ranging terms were used by researchers in these journals to describe ethnicity. Some used various aggregations of 'countries of birth' (e.g. North Asian, South East Asian, Asian), 'white' and 'non-white', 'non-English-speaking', or 'ethnic groupings', one defined ethnicity in a population as related to parental country of birth. This diversity of terms indicates the continued lack of healthcare researcher agreement about the appropriate ways of reporting ethnicity in Australia, although one study noted that government databases tend to follow nationally agreed terminology [100]. Few researchers outlined the study methods they used to assess the ethnicity-related variables. In articles that included some mention of multicultural health issues, few discussed the study's results related to ethnicity; the majority simply noted ethnicity as a demographic variable. There were few attempts in these studies to further analyse the effects of socio-economic status, gender, or educational status see Figure 3.

Datasets often contain little information on English language proficiency or on the demographic, social, economic and cultural factors that may influence health and health service utilisation [100,144]. Much class, ethnic and geographical variation in health status may be masked or homogenised because birthplaces or groups are aggregated (often in inconsistent ways) so as to achieve statistical meaning. At times, for example, all people born overseas are considered as one group, as are all people who speak a particular language, irrespective of their year of arrival, English language proficiency, age, and region of origin or socio-economic profile [144-147]. Bhopal refers to these epidemiological practices as 'inventing ethnic groups', 'lumping groups together', 'not adjusting for confounding factors' and 'not comparing like with like' [148]. Coming from a non-English-speaking country is frequently used as a surrogate for poor proficiency in English. This aggregation has an inherent bias as many immigrants from non-English-speaking countries actually speak very fluent English (e.g. immigrants from Hong Kong, India, Holland). Kliewer and Jones [144], in their study of newly arrived immigrants, found that almost 12% of immigrants from NESB countries spoke English as their preferred language and another 39% stated they spoke English 'well' or 'very well'. Fields that have been incorporated into standard healthcare databases, such as 'language spoken at home' or 'preferred language,' do not indicate how well a person speaks English.

The available research could be described as uneven in its coverage of major health status and health risk factor issues affecting immigrant communities. There are some major health status issues that appear to have received little attention; for example, renal disease, dialysis access, heart failure, kidney disease, prostate cancer, lung cancer, eye problems, cardiac problems, depression or chronic diseases. It is interesting that very few original articles dealt with patient experience, acute care, or the ethnic elderly. In terms of health promotion, the emphasis was limited to screening, general practice access, and interventions to promote population-based health screening.

Further, little attempt has been made to understand the crucial relationship between poor language proficiency, culture and patient safety in the Australian context. The question of whether there is ethnic disparity (difference in treatment and care based on ethnicity, race or language ability) in healthcare service provision has not been addressed in these major Australian healthcare journals.

Studies were mainly descriptive/observational studies or basic epidemiological studies. None were randomised control trials or longitudinal studies. Some studies were a series of cross-sectional surveys. There were no clinical trials. A few interventional studies looked at, for example, the effect of the ethnic media on service or screening access. While there were some studies with large samples, others had quite small numbers.

Interestingly, many ANZJPH articles studied the Vietnamese or 'South East Asian' populations, with 14 of the 20 articles which featured specific populations choosing to study South East Asians. Two other studies researched Arabic/Lebanese groups, two studied Italians, two studied Islanders, and one studied each of sub-Saharan Africans and Filipinas. Four studies were concerned with asylum seekers and a further three with refugees. This is an interesting representational bias even given that Vietnamese is one of the five major languages spoken in Australia. The reasons can only be speculated upon, but it is interesting to recall the words of Martin [8], who stated that in the 1950s and 1960s migrant groups were homogenised (as one) but that in the 1970s 'migrant' equalled Greek and Italian. The research data from the ANZJPH would seem to indicate that in the past decade, 'migrant' equals 'Vietnamese', 'South-East Asian', 'asylum seeker' or 'refugee'. This observation is also consistent with the representations of migrants in MJA original studies, with newly arrived refugees and asylum seekers being the major groups studied. It may be that by the 1990s and this 21st century, the term 'migrant' has come to mean South East Asian, Vietnamese, refugee and asylum seeker.

## Conclusions

The review of these three journals over a 12-year period demonstrates that the quantum, range and quality of the research and evidence which is required for equity in policy, services, interventions and implementation is limited. Kagawa-Singer [149] argued that mono-cultural health services' research focus has three major limitations, namely a lack of recognition of different world views, a lack of understanding of the most appropriate and effective means to cope with illnesses based on those world views, and a lack of ability to hear different ways of communicating these perspectives.

Whilst the MJA, AHR and ANZJPH research platforms could certainly not be called 'mono-cultural', they could quite reasonably be called 'limited' and 'uneven'. While there is some excellent quality research in respect of refugees, asylum seekers and Vietnamese immigrants, there are other communities and health issues that are essentially invisible or unrepresented in research.

## Methods

Systematic reviews were undertaken in three major Australian health care journals. These journals arguably represent the broad spectrum of mainstream healthcare research in Australia. The three journals include: the largest circulation general medical practice and clinical research publication in Australia, the *Medical Journal of Australia *(MJA), Australia's largest public health journal *The Australian and New Zealand Journal of Public Health *(ANZJPH) and Australia's major health services research and management journal, *The Australian Health Review *(AHR). Reviews were undertaken of the last twelve 12 years (1996 to August 2008) of journal articles using six standard search terms: 'non-English-speaking', 'ethnic', 'migrant', 'immigrant', 'refugee', and 'multicultural'. Each of the categorisations was tailored to the content of the journal. A single approach across these three journals was neither possible nor desirable.

Articles were categorised as being primarily concerned with multicultural health issues, having some level of inclusion of multicultural health issues or excluding multicultural health issues. Within the category of 'some inclusion', articles were further categorised a having a major, moderate or minor level of consideration of multicultural health issues. A major inclusion required that a large section or part of the argument of the article related to multicultural health or immigrant health issues; a moderate level of inclusion defined multicultural health or immigrant health issues as one of a range of issues within a broader context or set of issues. A minor inclusion meant that multicultural health or immigrant health was included in some minor way in the study, for example, as a demographic descriptor. A zero inclusion means that there was no consideration of multicultural or immigrant health issues in the article. The principal researcher undertook this categorisation. It was checked by a second researcher.

### Search strategy: *Medical Journal of Australia*

The MJA review strategy involved searching the six terms through the on-line ejournal. Articles were excluded if they were concerned with Aboriginal and Torres Strait Islander health, book reviews, repeat articles, advertisements, tables of content, or archives. Articles that did not contain the search words were not included. All other articles were included. A cross-check on this accession list was made via a verifying Medline and Scopus search. Articles were categorised as original research based on multicultural health issues or immigrant groups, original research that specifically excluded immigrant groups, original research that included multicultural health or immigrant health issues to some extent, medical workforce articles and clinical guidelines, policy, editorials and opinion pieces. The main search strategy and categorisation system is outlined in Figure 1.

### Search strategy: *Australian Health Review*

A similar process was undertaken using the same six keywords from 1996 to August 2008 in the *Australian Health Review *(AHR). AHR does not have an online search facility, so a search was undertaken using the Scopus database followed by a Medline search. The results were checked by a manual perusal of the articles. New Zealand articles were excluded. Articles were categorised as original research based on immigrant groups or multicultural health issues, original workforce articles, research methods and original research with some inclusion of immigrant groups. Articles were further categorised in terms of the extent of inclusion of immigrant groups or multicultural health. This search strategy is outlined in Figure 2.

### Search strategy: *Australian and New Zealand Journal of Public Health*

The third search was undertaken in the *Australian and New Zealand Journal of Public Health *(ANZJPH). Again, the Scopus search function was cross-checked with a Medline search. Articles about New Zealand health, Aboriginal and Torres Strait Islander health, Indigenous health, letters, book reviews, and repeat articles were excluded. Articles were categorised as original research based on immigrant groups or multicultural health and original research with some inclusion of immigrant groups or multicultural health. The original research based on immigrant groups was further categorised as policy articles, epidemiology or health status articles, interventional or methods studies, service or access articles, attitudinal or behaviour change articles and outcomes articles. Articles were further categorised in terms of the extent of inclusion of multicultural and immigrant health issues. This search strategy is outlined in Figure 3.

## Competing interests

The authors declare that they have no competing interests.

## Authors' contributions

PG: Designed and co-ordinated the study, reviewed all articles, interpreted and analysed findings and drafted the manuscript. HD: Participated in the conception and design of the study, contributed to the analysis, manuscript drafting and manuscript review. AW: Participated in the conception and design of the study, contributed to the analysis, manuscript drafting and manuscript review. LW: Carried out the literature searches, participated in the preliminary coverage analysis, and reviewed the manuscript.
